# Disability, quality of life and all-cause mortality in older Mexican adults: association with multimorbidity and frailty

**DOI:** 10.1186/s12877-018-0928-7

**Published:** 2018-10-04

**Authors:** Ana Rivera-Almaraz, Betty Manrique-Espinoza, José Alberto Ávila-Funes, Somnath Chatterji, Nirmala Naidoo, Paul Kowal, Aarón Salinas-Rodríguez

**Affiliations:** 1 National Institute of Public Health, Avenida Universidad #655, Colonia Santa María Ahuacatitlán, ZC, 62100 Cuernavaca, Morelos Mexico; 2 Department of Geriatrics, Salvador Zubirán National Institute of Medical Sciences and Nutrition, Mexico City, Mexico; 3 Department of Health Statistics and Information Systems, World Health Organization, Geneva, Switzerland; 4 University of Newcastle Research Centre on Gender, Health and Ageing, Newcastle, Australia; 5 Chiang Mai University Research Institute for Health Sciences, Chiang Mai, Thailand

**Keywords:** Multimorbidity, Frailty, Mortality, Disability, Quality of life, Older adults

## Abstract

**Background:**

Multimorbidity and frailty are relevant conditions among older adult population. There is growing evidence about their association with poor health outcomes like disability, worst quality of life, and death. Nonetheless, the independent associations of both conditions have been studied, and few evidence exists about an interaction between them. Our aims were to assess the association of frailty and multimorbidity with the disability, quality of life and all-cause mortality as well as to analyze a potential interaction between these conditions.

**Methods:**

Analytical samples included 1410 respondents for disability and quality of life, and 1792 for mortality. We performed a longitudinal analysis with older Mexican adults aged 50, using data collected from the WHO’s Study on global AGEing and Adult Health Waves 1 and 2. Disability was measured using the World Health Organization Disability Assessment Schedule (WHODAS 2.0), and quality of life using the WHOQOL (WHO Quality of Life) instrument. All-cause mortality was determined by reviewing death certificates. Associations of frailty and multimorbidity with disability, quality of life and mortality were estimated using linear regression and Cox proportional hazards models.

**Results:**

Multimorbidity assessed through three patterns (cardiopulmonary, vascular-metabolic, and mental-musculoskeletal) was associated with the three outcomes in this study. Cardiopulmonary and mental-musculoskeletal patterns increased the WHODAS mean score (β = 5.05; *p* < 0.01 and β = 5.10; *p* < 0.01, respectively) and decreased WHOQOL score (β = − 1.81; *p* < 0.01 and β = − 2.99; *p* < 0.01, respectively). Vascular-metabolic was associated with mortality (HR = 1.47; *p* = 0.04), disability (β = 3.27; *p* < 0.01) and quality of life (β = − 1.30; *p* = 0.02). Frailty was associated with mortality (pre-frail: HR = 1.48; *p* = 0.02 and frail: HR = 1.68; *p* = 0.03), disability (pre-frail: β = 5.02; *p* < 0.01; frail: β = 13.29; *p* < 0.01) and quality of life (pre-frail: β = − 2.23; *p* < 0.01; frail: β = − 4.38; *p* < 0.01). Interaction terms of frailty and multimorbidity were not statistically significant.

**Conclusions:**

Multimorbidity and frailty are important predictors of poor health outcomes. These results highlight the importance of carrying out health promotion and prevention actions as well as specific interventions aimed at older adults who suffer from multimorbidity and frailty, in such a way that deleterious effects on health can be avoided.

**Electronic supplementary material:**

The online version of this article (10.1186/s12877-018-0928-7) contains supplementary material, which is available to authorized users.

## Background

The proportion of the world’s population aged 60 and older is increasing rapidly. Within 35 years, it will have spiraled from 12% (in 2015) to 22% (in 2050). Virtually all the regions in the world are witnessing this population aging process [1]. From a public health perspective, older adults (OA) account for 23% of the global burden of disease, particularly as regards chronic diseases (CDs) such as cardiovascular, pulmonary, musculoskeletal, neurological, mental, and neoplastic conditions [2]. They have also been found to be at higher risk for multimorbidity and geriatric syndromes such as frailty [3].

Regarding multimorbidity, associations with greater use of health-care services and adverse health events such as polypharmacy, increased health expenditures, disability, a low quality of life, and even mortality have been reported [4–6]. However, multimorbidity research has traditionally focused on counting diseases, thus hindering the detection of co-occurrences. Notwithstanding, recent literature has highlighted the importance of taking into account the CDs combinations in OA health studies [7].

Frailty, on the other hand, defined as a biological syndrome resulting from cumulative declines across multiple physiologic systems, with impaired homeostatic reserve and a reduced capacity of the organism to withstand stress [8], has been identified as an independent predictor for adverse health outcomes including falls, a diminished quality of life, disability and death [9–14].

Although OA can suffer from multimorbidity and frailty simultaneously, a causal link has been suggested between them [15], empirical evidence has focused on the independent effects that they have on adverse health outcomes. Among the scarce evidence that has explored the joint effects of multimorbidity and frailty, one study with OA residents of Hong Kong found that combination of frailty and multimorbidity increased the risk of disability and death [16]. However, it is still pending to analyze this interaction effect on other outcomes such as health-related quality of life. Therefore, our main aim in this study was to estimate the independent associations of multimorbidity and frailty with three different outcomes: disability, quality of life and all-cause mortality. A secondary aim was to determine whether exist a significant interaction effect of the multimorbidity and frailty on those same outcomes.

## Methods

### Study design

We drew our data from the Study on global AGEing and adult health (SAGE), a longitudinal, multicenter study conducted by the World Health Organization (WHO) across six low- and middle-income countries: India, Ghana, Russia, Mexico, South Africa and China [17]. Based on a multistage, stratified and cluster design, SAGE provided nationally representative groups of adults aged 50 and older, disaggregated by rural/urban strata. It applied standardized instruments including household and individual-level questionnaires, as well as anthropometric and functionality measurements. The present study consisted of a longitudinal analysis of SAGE-Mexico data derived from the two measurements available to date (2009 and 2014).

### Study sample

#### Baseline measurement

In 2009, Wave 1 performed baseline measurements with a sample size of 2306 adults aged 50 years and older and a smaller comparison group of 436 subjects aged 18–49 years.

#### Follow-up

The first follow-up (Wave 2), conducted in 2014, included all eligible subjects from Wave 1. In addition, the sample was refreshed with a sample of both age groups: one from 18 to 49 years, the other 50 years and older. A total response rate of 83.47% was achieved. Full interviews were administered to a larger number of participants than in Wave 1: 853 aged 18–49 years, and 4301 aged 50 and older.

**Fig. 1:**
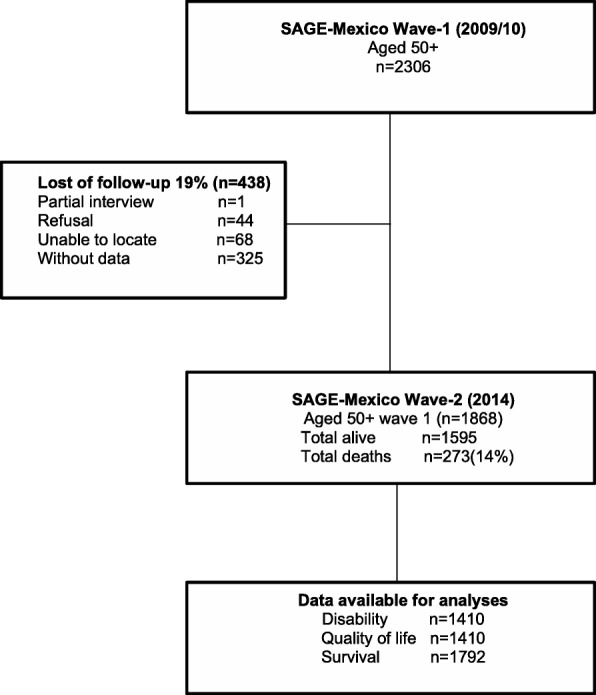
Population study and analytical sample

Our analytical sample included Wave 1 respondents aged 50 and older as well as Wave 2 subjects who provided information on the variables of interest. Of the 2306 OA in Wave 1, 1595 were interviewed and 273 had died (14.61%). The remaining 438 (19%) either declined our invitation to participate in the study or were not located. Analytical samples of OA aged 50 and older included 1410 respondents for disability and quality of life, and 1792 for mortality (Fig. 1).

Outcomes. We analyzed three of the Wave 2 outcomes: disability, quality of life and all-cause mortality.

##### Disability

We measured this variable according to the World Health Organization Disability Assessment Schedule (WHODAS 2.0) [18]. The WHODAS 2.0 scale is widely used to measure last-month limitations in activity and daily-life participation. It covers six domains explored through a total of 12 items (two per domain): (1) cognition and communication, (2) self-care, (3) mobility, (4) interpersonal relations, (5) life activities, and (6) participation. The results of the 12 items are summed up to obtain a global score expressed on a continuous scale from 0 (no disability) to 100 (full disability) [18].

##### Quality of life

For this variable, we used the WHOQOL (WHO Quality of Life) instrument. This eight-item questionnaire covers the following core domains (two items per domain): physical, psychological, social and environmental. The results of the eight items are added up to obtain an overall WHOQOL score expressed on a scale from 0 to 100. The higher the score, the higher the quality of life [19].

##### All-cause mortality

Data drawn from the Wave 2 interviews (2014) included information about death (for any cause) occurred between Waves 1 and 2 using a verbal autopsy. We recorded the date of death, which provided information on the interval between baseline and death.

### Exposures

#### Multimorbidity

We used a nine CDs list that was included in the SAGE study. Three were measured according to self-reported medical diagnoses: diabetes, stroke, and cataracts. Five were estimated through algorithms for symptomatology and self-reported treatment: angina, arthritis, chronic obstructive pulmonary disease, asthma, and depression [20]. Finally, hypertension was determined by either blood pressure measurement and/or self-reported treatment. The CDs were assessed dichotomously (1 = presence, 0 = absence).

#### Frailty

For this variable, we used the frailty phenotype proposed by Fried et al. (2001), which covers five components: unintended weight loss, exhaustion, low physical activity, slow walking speed and weakness. Subjects considered frail presented three or more of these components; pre-frail one or two; and not frail, or robust, none. The results were thus expressed categorically: 0 = not frail, 1 = pre-frail, and 2 = frail [8]. Frailty components were assessed as follows:

1. *Unintended weight loss* during the past year *was* determined according to a self-reported reduction of five kilograms or more in the past 12 months. Subjects with this condition were considered frail based on weight loss.
2. *Exhaustion* was assessed according to a question from the World Health Organization World Mental Health Composite International Diagnostic Interview (WHO WMH-CIDI). The question took into account the definition and criteria for “depressive episode” diagnosis as specified in the International Statistical Classification of Diseases and Related Health Problems (ICD-10) and in the Diagnostic and Statistical Manual of Mental Disorders (DSM IV). It was used as a proxy for the depression-scale questions in the Center for Epidemiologic Studies Depression Scale (CES-D) used by Fried et al. in their definition of exhaustion. Subjects were specifically asked, “During the last 12 months, have you had a period lasting several days when you have been feeling your energy decreased or that you are tired all the time?” Those who gave a positive response were considered frail based on exhaustion.
3. *Low physical activity* was determined according to the Global Physical Activity Questionnaire (GPAQ). It was measured by estimating the weekly caloric expenditure of respondents, stratified by sex. Those who fell within the fifth lowest percentile of weekly kilocalories, by sex, were considered frail based on physical activity.
4. *Slow walking speed* was determined according to the time (seconds) it took participants to walk a distance of four meters at normal speed. Slow walking speed corresponded to a time span equal to or greater than the 80th percentile (in seconds), stratified by sex and median height.
5. *Weakness* was measured with a dynamometer. Results were stratified by sex and quartiles of body mass index (BMI). We first considered maximum grip strength values, and then obtained quintiles for each BMI quartile. Low grip strength was equivalent to the lowest quintile in each BMI quartile by sex.

### Covariates

Using the literature on factors associated with older adults’ mortality and disability as a guide, risk factors were selected as potential covariates and then identified in the SAGE dataset. Covariates were categorized as follows: age, sex (1 = female), education (years of schooling), marital status (1 = with partner), place of residence (1 = rural), and household income using a continuous index based on household assets, where higher positive values indicated higher incomes.

## Statistical analysis

We used bivariate analyses to examine the relationships between the independent variables (frailty and multimorbidity patterns) and the dependent variables (disability, quality of life and mortality). We employed the following tests: Chi-square for categorical variables and ANOVA or Kruskall-Wallis for continuous variables.

### Multimorbidity patterns

As we were interested in the CDs combinations more than a simple count of them, we performed a principal-components analysis of the nine CDs mentioned above to identify possible multimorbidity patterns. Given that the CDs were coded dichotomously, we built a polychoric correlation matrix, with the optimal number of patterns (components) determined according to the rule of eigenvalues greater than one. Then each CD was assigned to the component where its coefficient yielded the highest factor loading (score).

### Disability and quality of life

To assess the relationships of the multimorbidity patterns and frailty with (1) disability and (2) quality of life, we applied linear regression models.

### Mortality

To analyze mortality, we explored participant survival time based on the Cox proportional hazards model. We performed exploratory analysis by estimating Kaplan-Meier curves for all categorical predictors, and log-rank test for equality of strata to assess the predictors in the final model. We selected model predictors according to the exploratory analysis and prior evidence on OA survival.

Finally, we assessed the interaction effects between frailty and each multimorbidity patterns. We tested the statistical significance of this potential interaction effect by including a multiplicative term between frailty and multimorbidity patterns into the three regression models.

Data were weighted using post-stratified individual probability weights based on the selection probability at each stage of selection. Differences were considered statistically significant if *p* < 0.05. All statistical analyses were performed using STATA version 15.1 software (StataCorp. 2017. College Station, TX.).

## Results

### Baseline characteristics

Table 1 presents the distribution of the study variables at baseline. Mean scores for WHODAS 2.0 and WHOQOL were 15.7 (SD = 16.3) and 47.4 (SD = 11.8) respectively. Meanwhile, prevalence rates for multimorbidity patterns were: 14.5% for cardiopulmonary, 68.3% for vascular-metabolic, and 21.5% for mental-musculoskeletal. Frailty prevalence was 7.9% and for pre-frailty was 58.0%.

**Table 1:** Baseline health and socio-demographic characteristics

	Mean/Percentage	S.D.
Outcomes		
Disability (WHODAS 2.0)^a^	15.7	16.3
Quality of life (WHOQOL)^b^	47.4	11.8
Main exposure variables		
Multimorbidity	34.5	
Multimorbidity patterns		
Pattern 1: Cardiopulmonary^c^	14.5	
Pattern 2: Vascular-metabolic^d^	68.3	
Pattern 3: Mental-musculoskeletal^e^	21.5	
Frailty		
Nonfrail	34.1	
Prefrail	58.0	
Frail	7.9	
Covariates		
Age (years)	63.0	10.6
Female	53.2	
Marital status (with partner)	73.0	
Education (years)	5.0	4.5
Residence (rural)	21.2	
Income	0.09	0.41

^a^WHODAS 2.0 (0 = no disability, 100 = total disability); ^b^WHOQOL (0 = worst quality of life, 100 = best quality of life); ^c^Pattern 1: chronic obstructive pulmonary disease, asthma and angina; ^d^Pattern 2: diabetes, hypertension, stroke and cataracts; ^e^Pattern 3: arthritis and depression

### Multimorbidity patterns

We identified three multimorbidity patterns that explained 55% of the variance among the nine CDs analyzed. Pattern 1, cardiopulmonary conditions, included angina, chronic obstructive pulmonary disease, and asthma; Pattern 2, metabolic-vascular conditions, included diabetes, arterial hypertension, stroke and cataracts; and Pattern 3, mental-musculoskeletal conditions, included arthritis and depression (see Additional file 1: Figure S1 and Table S1).

Table 2 displays the bivariate associations of frailty and multimorbidity with disability, quality of life and all-cause mortality.

**Table 2:** Bivariate associations of frailty and multimorbidity with disability, quality of life and all-cause mortality

Main exposure variables	Disability (WHODAS 2.0)^a^ *n* = 1410		Quality of life (WHOQOL)^b^ *n* = 1410		Mortality *n* = 1792	
	Mean	*p value*	Mean	*p value*	Percentage	*p value*
Frailty						
Nonfrail	8.7	*< 0.001*	56	*< 0.001*	4.3	*< 0.001*
Prefrail	19.4		51.1		10.2	
Frail	26.1		51.5		29.6	
Multimorbidity patterns						
Pattern 1: Cardiopulmonary^c^						
Presence	20.9	*< 0.001*	50.2	*< 0.001*	10.4	*0.966*
Absence	14.6		53.5		9.7	
Pattern 2: Vascular-metabolic^d^						
Presence	14.8	*< 0.001*	53.4	*0.004*	12.8	*< 0.001*
Absence	16.8		52.2		3.6	
Pattern 3: Mental-musculoskeletal^e^						
Presence	21.9	*< 0.001*	46	*< 0.001*	6.5	*0.751*
Absence	13.6		55.1		10.8	

^a^WHODAS 2.0 (0 = no disability, 100 = total disability); ^b^WHOQOL (0 = worst quality of life, 100 = best quality of life); ^c^Pattern 1: chronic obstructive pulmonary disease, asthma and angina; ^d^Pattern 2: diabetes, hypertension, stroke and cataracts; ^e^Pattern 3: arthritis and depression

#### Disability

The presence of frailty was associated with disability, pre-frail and frail subjects indicating a higher average WHODAS score (*p* < 0.001). As regards multimorbidity patterns, cardiopulmonary conditions (*p* < 0.001) and mental-musculoskeletal conditions (*p* < 0.001) were also associated with greater disability.

#### Quality of life

Lower quality of life was observed in pre-frail and frail subjects (*p* < 0.001). Furthermore, the presence cardiopulmonary conditions (*p* < 0.001) or mental-musculoskeletal conditions (*p* < 0.001) was associated with lower WHOQOL scores.

#### Mortality

Mortality was greater among frail (29.6%) and pre-frail (10.2%) OA as compared to those who were not frail (4.3%) (*p* < 0.001). Those exhibiting metabolic-vascular conditions had a higher mortality rate in comparison with those not suffering from these conditions: 12.8% versus 3.6% (< 0.001).

Table 3 shows the results of three regression models used to estimate the association between our main exposures (frailty and multimorbidity) and the three outcomes: disability, quality of life, and mortality.

**Table 3:** Adjusted associations of frailty and multimorbidity with disability, quality of life and all-cause mortality

	Model 1 Disability (WHODAS 2.0)^a^		Model 2 Quality of life (WHOQOL)^b^		Model 3 Mortality	
	β	(95% CI)	β	(95% CI)	HR	(95% CI)
Frailty						
Nonfrail	Reference		Reference		Reference	
Prefrail	5.02	(3.24,6.80)*	−2.23	(−3.23,-1.24)*	1.48	(1.05,2.09)*
Frail	13.29	(9.81,16.78)*	−4.38	(−6.32,-2.43)*	1.68	(1.06,2.68)*
Multimorbidity patterns						
Pattern 1: Cardiopulmonary^c^	5.05	(2.86,7.25)*	−1.81	(−3.04,-0.58)*	0.82	(0.58,1.14)
Pattern 2: Vascular-metabolic^d^	3.27	(1.40,5.13)*	−1.3	(−2.34,-0.26)*	1.47	(1.03,2.10)*
Pattern 3: Mental-musculoskeletal^e^	5.1	(2.98,7.22)*	−2.99	(−4.17,-1.81)*	0.72	(0.52,1.01)

*p < 0.05; β: beta coefficient; HR: hazard ratio; CI: confidence interval. ^a^WHODAS 2.0 (0 = no disability, 100 = total disability); ^b^WHOQOL (0 = worst quality of life, 100 = best quality of life). ^c^Pattern 1: chronic obstructive pulmonary disease, asthma and angina; ^d^Pattern 2: diabetes, hypertension, stroke and cataracts; ^e^Pattern 3: arthritis and depression. All models were adjusted for age, sex, marital status, education (years), residence, and household income; only the Cox proportional model for mortality risk was adjusted for these variables plus disability (WHODAS 2.0 score)

Regarding disability, the results showed that pre-frailty and frailty increased the level of disability. Compared to non-frail, WHODAS score increased five points among pre-frail (β = 5.02; 95% CI 3.24; 6.80), and 13 points for frails individuals (β = 13.29; 95% CI 9.81; 16.78). For multimorbidity patterns, OA with cardiopulmonary and mental-musculoskeletal conditions had an increase in WHODAS scores (β = 5.05, and β = 5.10, respectively) and those with vascular-metabolic conditions had a slightly minor increase (β = 3.27) (Model 1).

For the quality of life, pre-frail and frail individuals had lowest scores in WHOQOL than non-frail subjects (β = − 2.2, and β = − 4.3, respectively). Similarly, the three morbidity patterns diminished the mean WHOQOL scores. Musculoskeletal conditions had the greatest effect (β = − 2.99; 95% IC -4.17;-1.81), meanwhile cardiopulmonary conditions (β = − 1.81, 95% CI -3.04,-0.58), and vascular-metabolic conditions (β = − 1.30, 95% CI -2.34,-0.26) had a moderately minor impact (Model 2).

**Fig. 2:**
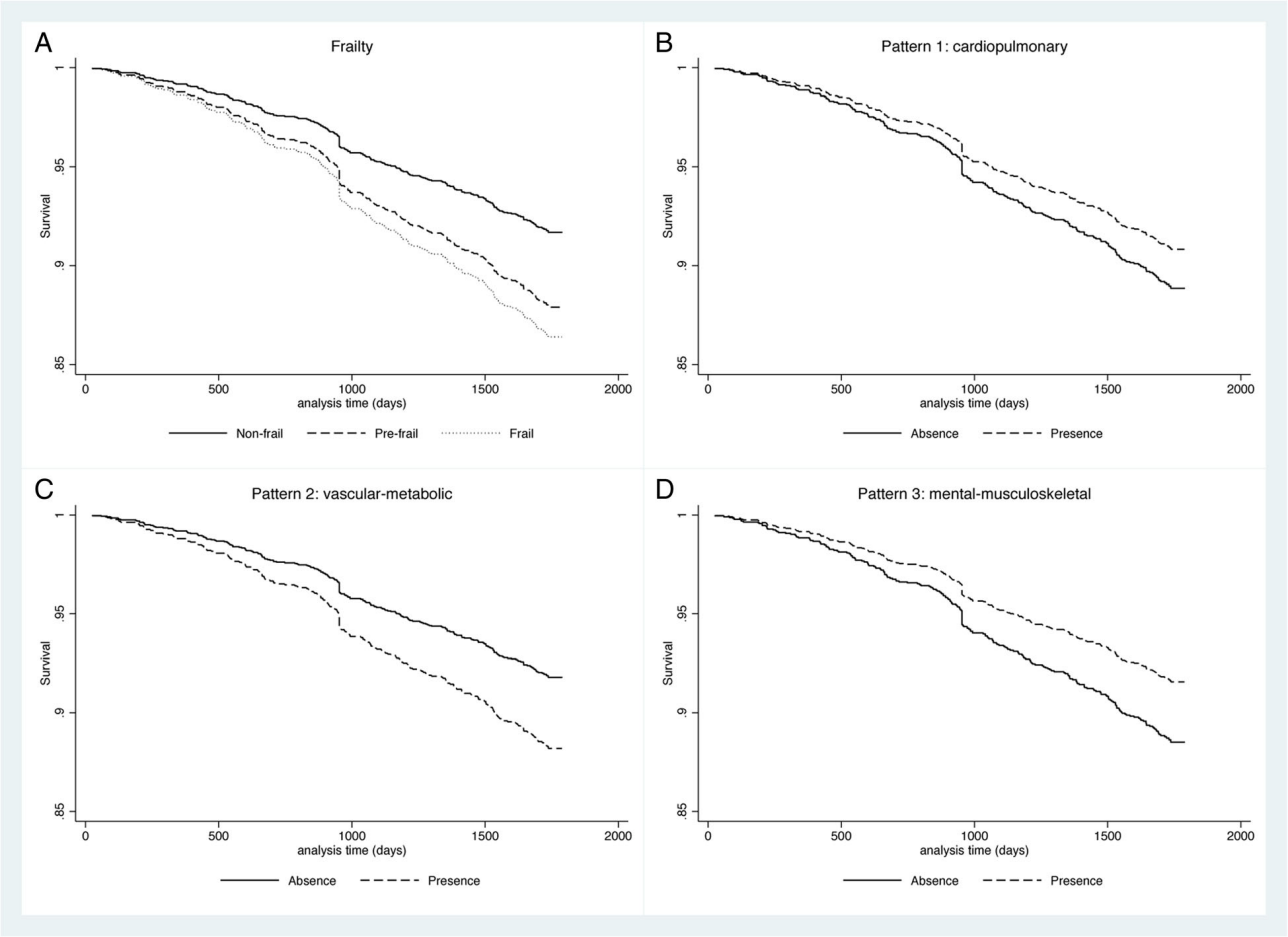
Survival function for the Cox proportional hazard regression by (**a**) frailty categories (**b**) cardiopulmonary pattern; (**c**) vascular-metabolic pattern; and (**d**) mental-musculoskeletal pattern

Related to mortality, results of the Cox models showed that pre-frail OA had a risk of death 48% higher than that non-frail (HR = 1.48; 95% CI 1.05; 2.09), and for the frail subjects that risk was 68% higher (HR = 1.68; 95% CI 1.06; 2.68). For the three morbidity patterns analyzed, only vascular-metabolic condition was associated with an increased risk of death (HR = 1.47; 95% CI 1.03; 2.10) (Model 3). Figure 2 depicts graphically the results of the Cox models for our two exposures, frailty and multimorbidity patterns.

Finally, results showed that none interaction term (between frailty and multimorbidity patterns) proved statistically significant (see Additional file 2: Table S2).

## Discussion

The results of this study show that frailty and multimorbidity were independently associated with the disability, quality of life and mortality after 5-years follow-up among a national sample of older Mexican adults. Frailty and multimorbidity rose the mean scores of the WHODAS and WHOQOL and also increased the risk of death. However, no significant interactions between frailty and multimorbidity were found.

Our study identified three multimorbidity patterns in Mexican OA, which were: cardiopulmonary, vascular-metabolic and mental-musculoskeletal. The evidence related to multimorbidity patterns has led us to conclude that these patterns are composed of diseases sharing a number of similarities from a clinical perspective.

Regarding the first pattern, cardiopulmonary condition, comprised of asthma, chronic obstructive pulmonary disease (COPD), and angina, a clinical and molecular link had been previously established [21, 22]. For the second pattern identified, vascular-metabolic conditions, evidence support the relationship between metabolic syndrome (chronic inflammation, adiposity, etc.) and aging. This pattern includes CDs such as diabetes, arterial hypertension, stroke and cataracts [23, 24]. Finally, the third pattern, comprising arthritis and depression (mental-musculoskeletal), can be explained by the link reported several decades ago between pain and mental illness, which suggests that they share a number of biological pathways [25–27].

Our findings indicate that the three multimorbidity patterns analyzed were independent predictors for increased disability among OA. These results are consistent with the studies that have analyzed this link taking multimorbidity patterns into account. Jackson et al. (2015) studied a cohort of older Australian women and found that patterns similar to mental-neurological and cardiopulmonary conditions were associated with higher levels of disability (measured by the basic and instrumental activities of daily life - ADL and IADL, respectively) [28]. Furthermore, Arokiasamy et al. reported an association between diabetes and hypertension combination and the presence of disability (+ 1 ADL) in adults 18 and older [5]. Otiniano et al. (2003) found that the combination of diabetes and stroke increased the risk of disability (1 + ADL and 1+ IADL) in Mexican-American OA aged 65 and older, at five-year follow-up [29]. Quinones et al. (2016) reported that the combination of arthritis, depression and hypertension in American OA was associated with higher levels of disability (combined ADL and IADL index) at two-year follow-up [30].

Frailty was also an independent predictor for disability. This finding is also consistent with those reported in the literature [9, 31], even allowing for different follow-up periods [8, 10, 32, 33]. In the Mexican context, at 11-year follow-up, the cohort of OA aged 60 years and older from the Mexican Health and Aging Study (MHAS) indicated that frailty was a predictor for disability as regards ADL, but not IADL, while pre-frailty was a predictor only for restricted mobility, but not for ADL or IADL [9]. Another study of urban OA aged 70 and older in the Mexican Coyoacan Cohort Study found that frailty increased ADL and IADL disability [31].

In our study disability was measured using the WHODAS 2.0, which traditionally has been done using ADL and IADL criteria in older adult population. The use of WHODAS allowed that functionality spectrum of OA was enhanced [34]. This means that vulnerability resulting from frailty in OA could affect other spheres beyond the physical dimension, inter alia, their interaction with others and their participation in society.

We also found that frailty and multimorbidity diminished the quality of life as measured in the follow-up study. These results are consistent with those of Arokiasamy et al., who reported that the combination of asthma and hypertension correlated with a diminished quality of life in adults aged 18 and older [5]. Moreover there is evidence that depression among OA is independently associated with a lower quality of life [35], and that adults with arthritis have a particularly high probability of suffering from a deteriorated quality of life [36].

Our results indicate that baseline frailty status were an independent predictor for a deteriorated quality of life in the follow-up. There is scarce longitudinal evidence of an association between frailty and quality of life [13, 37]. Even so, it has been hypothesized that this association could be bidirectional, baseline frailty could turns out to be predictive of a deteriorated quality of life, just as a low quality of life at baseline could be a predictor of frailty at follow-up [37]. Our results appears to provide support for the first scenario, which means that a decreased functionality among frail OA affects their satisfaction in various areas (physical and social) that are measured with instruments such as the WHOQOL [12, 13, 37]. However, this association must be deeply explored in future longitudinal studies with older adults.

Related to frailty and mortality, our results were consistent with previous studies that have identified frailty is an independent predictor for death. A systematic review and meta-analysis of longitudinal studies with OA using the frailty phenotype, found that frail subjects had a risk of dying two times higher than non-frail subjects. Pre-frailty also increased the risk of death, although the association was weaker [38]. For older Mexican adults, this association was also reported by various studies. Specifically, Mexican Health and Aging Study (*MHAS*) and 10/66 Dementia Study found that frailty was a predictor for death [9, 39].

Observed association between the metabolic-vascular pattern and mortality, supports the reported evidence on the relationship between metabolic syndrome and the likelihood of die [40], although recently it has been suggested that this association can be mediated by factors such as frailty [41, 42] and sleep disorders [43]. Nevertheless, even controlling for frailty, disability and other variables, it has been found that the metabolic-vascular combination has an independent effect on survival rate among OA.

A secondary objective of this study was to evaluate the potential interaction effects of multimorbidity and frailty. Although we did not find a significant interaction between these conditions, evidence suggests that a causal connection could exist, given they share common physiopathological mechanisms [15]. Even so, few studies have explored the possibility of a combined effect. Among them, Woo et al. (2014) found that combination of frailty and multimorbidity increased the risk of disability and death in OA [16]. Future research with OA that deeply explore on different combinations of chronic conditions will help to understand the potential interaction of frailty and multimorbidity and their effects on diverse health outcomes.

The results of our study should be interpreted taking into account the following limitations. First, the analysis of multimorbidity was confined to the nine high burden chronic conditions utilized in SAGE; it did not include diseases such as chronic kidney failure, cancer, cardiac conditions and dyslipidemia, all prevalent in older Mexican adults. Nevertheless, various studies using larger, equal or lower number of CDs, have found similar multimorbidity patterns. Second, potential selection bias may have resulted from differences between the analytical sample and excluded OA. Respondents in the study proved somewhat more affluent than respondents excluded (see Additional file 2: Table S3), it is not clear how this could affect our results, although it is possible that people with a higher economic level have greater knowledge about their health status, and then our prevalence of multimorbidity patterns could be underestimated. If the above were true, then our associations could be somewhat biased.

## Conclusion

Our findings are consistent with previous evidence about the effects of frailty and multimorbidity on disability, quality of life, and risk of death. These results highlight the importance of carrying out health promotion and prevention actions as well as specific interventions aimed at older adults who suffer from multimorbidity and frailty, in such a way that deleterious effects on health can be avoided. Although a potential interaction effect between frailty and multimorbidity was hypothesized, this association was not statistically significant.

## Additional files

Additional file 1: **Figure S1.** Screeplot for multimorbidity patterns. **Table S1.** Eigenvectors values for the three multimorbidity patterns identified. (PPTX 75 kb)

Additional file 2: **Table S2.** Interaction effects of multimorbidity patterns and frailty on disability, quality of life and mortality. **Table S3.** Observed differences between older adults included and excluded from the study. (DOCX 24 kb)

## Abbreviations

ADL: Basic activities of daily life
BMI: Body mass index
CDs: Chronic diseases
CES-D: Center for Epidemiologic Studies Depression Scale
CI: Confidence Interval
COPD: Chronic obstructive pulmonary disease
DSM IV: Diagnostic and Statistical Manual of Mental Disorders
GPAQ: Global Physical Activity Questionnaire
HR: Hazard Ratio
IADL: Instrumental activities of daily life
ICD-10: International Statistical Classification of Diseases and Related Health Problems
MHAS: Mexican Health and Aging Study
NS: Not Statistically significant
OA: Older adults
SAGE: Study on global AGEing and adult health
SF-36: 36-Item Short Form Health Survey
WHO WMH-CIDI: World Health Organization World Mental Health Composite International Diagnostic Interview
WHO: World Health Organization
WHODAS 2.0: World Health Organization Disability Assessment Schedule 2.0
WHOQOL: WHO Quality of Life
β: Beta Coefficient
