# Supplementary material for: Disability, quality of life and all-cause mortality in older Mexican adults: association with multimorbidity and frailty
Source: BMC Geriatr. 2018 Oct 4;18:236. doi: 10.1186/s12877-018-0928-7 (PMC6172837; doi:10.1186/s12877-018-0928-7)
Supplement: Supplementary file 1 — Figure S1. Screeplot for multimorbidity patterns. Table S1. Eigenvectors values for the three multimorbidity patterns identified. (PPTX 75 kb) [file 12877_2018_928_MOESM1_ESM.pptx]

## Slide 1
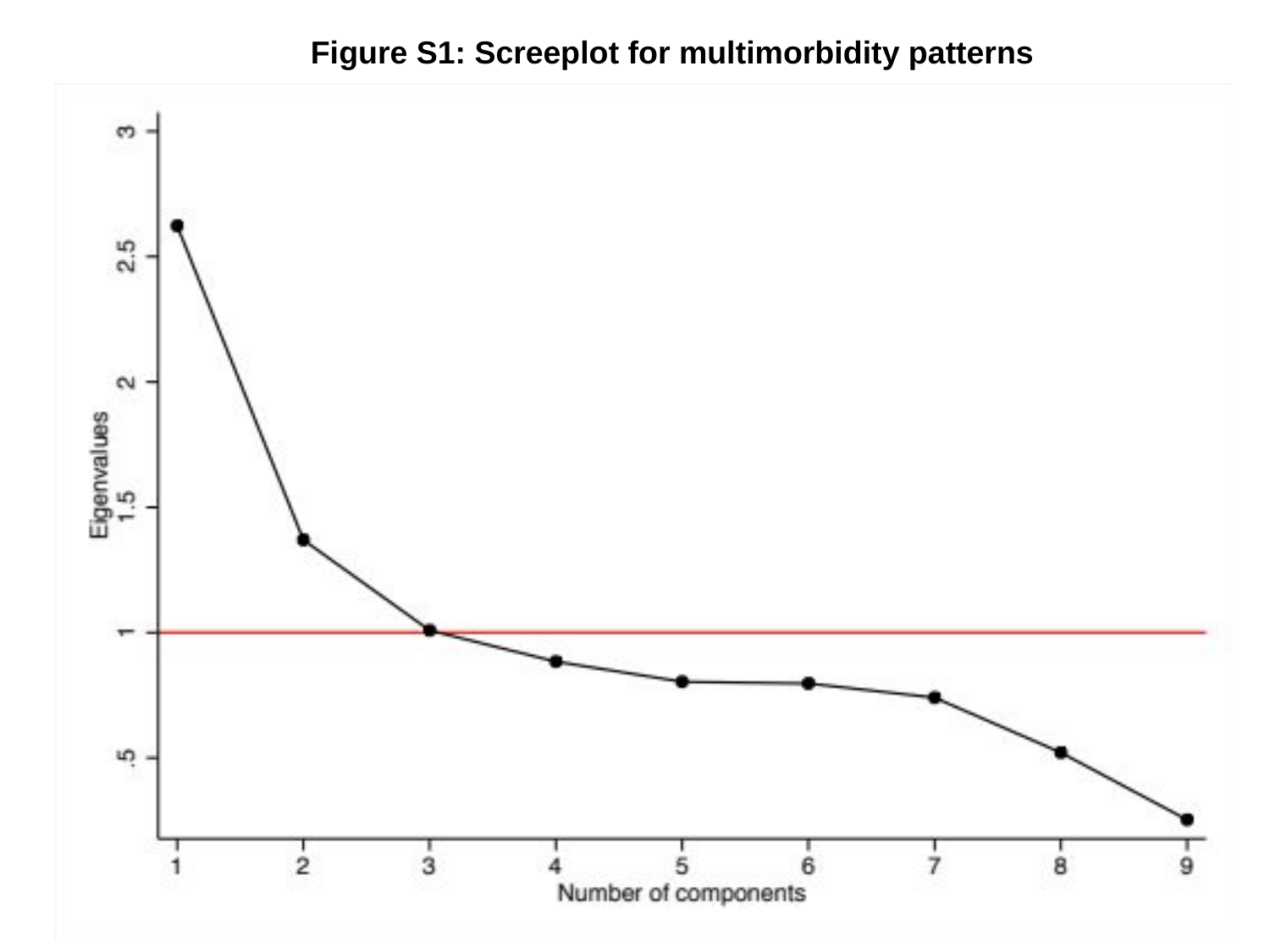

Figure S1: Screeplot for multimorbidity patterns

## Slide 2
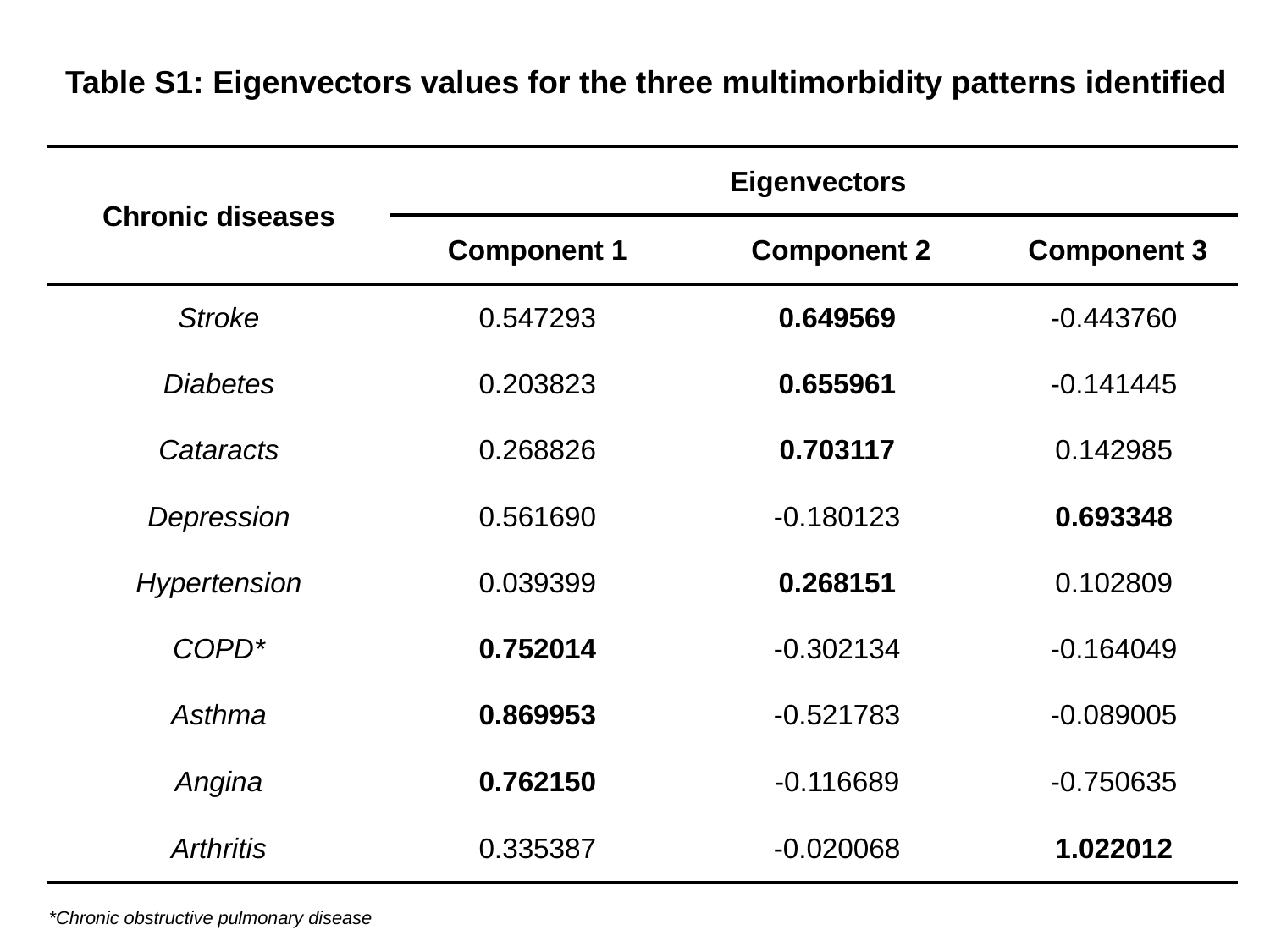

Table S1: Eigenvectors values for the three multimorbidity patterns identified
| Chronic diseases | Eigenvectors | | |
| --- | --- | --- | --- |
| | Component 1 | Component 2 | Component 3 |
| Stroke | 0.547293 | 0.649569 | -0.443760 |
| Diabetes | 0.203823 | 0.655961 | -0.141445 |
| Cataracts | 0.268826 | 0.703117 | 0.142985 |
| Depression | 0.561690 | -0.180123 | 0.693348 |
| Hypertension | 0.039399 | 0.268151 | 0.102809 |
| COPD\* | 0.752014 | -0.302134 | -0.164049 |
| Asthma | 0.869953 | -0.521783 | -0.089005 |
| Angina | 0.762150 | -0.116689 | -0.750635 |
| Arthritis | 0.335387 | -0.020068 | 1.022012 |
| \*Chronic obstructive pulmonary disease | | | |
