# Supplementary material for: Disability, quality of life and all-cause mortality in older Mexican adults: association with multimorbidity and frailty
Source: BMC Geriatr. 2018 Oct 4;18:236. doi: 10.1186/s12877-018-0928-7 (PMC6172837; doi:10.1186/s12877-018-0928-7)
Supplement: Supplementary file 2 — Table S2. Interaction effects of multimorbidity patterns and frailty on disability, quality of life and mortality. Table S3. Observed differences between older adults included and excluded from the study. (DOCX 24 kb) [file 12877_2018_928_MOESM2_ESM.docx]

**Table S2: Interaction effects of multimorbidity patterns and frailty on disability, quality of life and all-cause mortality**

|  | Disability (WHODAS 2.0)^a^ | | Quality of life (WHOQOL)^b^ | | Mortality | |
| --- | --- | --- | --- | --- | --- | --- |
|  | β | *p value* | β | *p value** | HR | *p value* |
| Pattern 1: Cardiopulmonary^c^ |  |  |  |  |  |  |
| Nonfrail | -1.22 | 0.522 | 0.06 | 0.958 | 0.71 | 0.475 |
| Prefrail | 3.30 | 0.604 | -0.79 | 0.529 | 0.85 | 0.726 |
| Frail | 2.52 | 0.326 | -0.41 | 0.833 | 0.79 | 0.861 |
| Pattern 2: Vascular-metabolic^d^ |  |  |  |  |  |  |
| Nonfrail | 0.62 | 0.647 | -0.51 | 0.521 | 1.41 | 0.338 |
| Prefrail | 5.07 | 0.144 | -1.67 | 0.263 | 1.45 | 0.939 |
| Frail | -4.10 | 0.273 | -1.06 | 0.826 | 1.67 | 0.790 |
| Pattern 3: Mental-musculoskeletal^e^ |  |  |  |  |  |  |
| Nonfrail | -0.01 | 0.996 | 1.03 | 0.383 | 0.85 | 0.725 |
| Prefrail | 3.26 | 0.166 | -3.01 | 0.060 | 0.71 | 0.731 |
| Frail | 4.45 | 0.198 | -3.29 | 0.399 | 0.70 | 0.743 |

β: beta coefficient; HR: hazard ratio; ^a^WHODAS 2.0 (0=no disability, 100=total disability); ^b^WHOQOL (0=worst quality of life, 100=best quality of life). ^c^Pattern 1: chronic obstructive pulmonary disease**,**  asthma and angina; ^d^Pattern 2: diabetes, hypertension, stroke and cataracts; ^e^Pattern 3: arthritis and depression. All models were adjusted for age, sex, marital status, education (years), residence, and household income; only the Cox proportional model for mortality risk was adjusted for these variables plus disability (WHODAS 2.0 score)

**Table S3:** **Observed differences between older adults included and excluded from the study**

|  | **Older adults included (n=1792)** | **Older adults excluded (n=514)** |  |
| --- | --- | --- | --- |
| **Measures in 2009** | **Mean (S.D.)/Percentage** | **Mean (S.D.)/Percentage** | ***p value*** |
| Multimorbidity patterns |  |  |  |
| Pattern 1: Cardiopulmonary^a^ | 16.1 | 9.2 | 0.250 |
| Pattern 2: Vascular-metabolic^b^ | 66.7 | 73.4 | 0.763 |
| Pattern 3: Mental-musculoskeletal^c^ | 23.0 | 16.5 | 0.947 |
| Frailty |  |  |  |
| Nonfrail | 36.0 | 28.3 | 0.730 |
| Prefrail | 55.4 | 66.0 |  |
| Frail | 8.7 | 5.7 |  |
| Age (years) | 62.8 (10.2) | 63.6 (11.4) | **<0.001** |
| Female | 54.9 | 48.1 | 0.720 |
| Marital status (with partner) | 73.3 | 72.0 | **0.002** |
| Education (years) | 4.6 (4.5) | 6.3 (4.1) | **<0.001** |
| Residence (rural) | 24.9 | 10.4 | **<0.001** |
| Income | 0.07 (0.42) | 0.15 (0.38) | **0.0125** |

^a^Pattern 1: chronic obstructive pulmonary disease, asthma and angina; ^b^Pattern 2: diabetes, hypertension, stroke and cataracts; ^c^Pattern 3: arthritis and depression; S.D. standard deviation
